# Riboflavin-primed seeds reduce arsenic stress in *Helianthus annuus* L. through modulating antioxidant and metabolomics responses

**DOI:** 10.7717/peerj.21596

**Published:** 2026-10-06

**Authors:** Anum Manzoor, Atif Kamran, Amna Naveed, Summera Jahan, Waheed Akram, Rehana Sardar, Nasim Ahmad Yasin

**Affiliations:** 1 Institute of Botany, University of the Punjab, Lahore, Pakistan; 2 Department of Plant Pathology, University of the Punjab, Lahore, Pakistan; 3 Department of Biological and Environmental Sciences, Emerson University, Multan, Pakistan; 4 Department of Horticulture, University of the Punjab, Lahore, Pakistan

**Keywords:** *Helianthus annuus*, Vitamin-priming, Antioxidant system, GC-MS, Metal toxicity, Stress alleviation

## Abstract

Heavy metals-induced stress disrupts plant growth and development, making their survival difficult. Arsenic (As) stress is one of the major constraints that have become obstacles to the successful cultivation of many oilseed plants, especially sunflower (*Helianthus annuus* L.). The current study aimed to estimate the capacity of riboflavin (Rib) to mitigate As stress in *H. annuus*. *H. annuus* seeds were primed with Rib (50 ppm) and grown in soil contaminated with As at 10 ppm per kilogram. Results demonstrated that As-stress negatively affected plant growth by increasing malondialdehyde (MDA) by 138.32% and electrolyte leakage to 54.43%. Stressed plants exhibited reduced antioxidant enzyme activities, reduced plant height to 40.5%, impaired gaseous exchange, and decreased photosynthesis to 22.30%, whereas Rib seed priming alleviated As toxicity by ameliorating membrane stability (from 45.57% to 55.80%), gas exchange, and enzymatic antioxidant activity (CAT, POD, APX) in treated plants. Moreover, Rib reduced MDA levels and increased chlorophyll content. The GC/MS-based non-targeted metabolic profiling indicated that seed priming with Rib could cause shifts in metabolites including sugars, amino acids, alcohols, carboxylic acids, and organic acids to augment *H. annuus* survival against metal stress. Our study demonstrates that seed priming with Rib can improve plant growth under metal stress in conventional agricultural systems.

## Introduction

The sustainability of the agricultural ecosystem is compromised by environmental pollution with metals, ultimately causing deleterious effects not only on living organisms but also on plants (Faizan et al., 2025). Among several heavy metals, arsenic (As) is widespread due to the use of agrochemicals and the disposal of untreated industrial effluents (Thakur et al., 2021). Arsenic contamination is a crucial problem for wastewater-irrigated lands (Natasha et al. 2021). According to a report published by the World Health Organization, around 200 million people are at risk of exposure to As toxicity, with the recommended level of As in drinking water set at 10 µg L^−1^ (WHO, 2022).

The Environmental Protection Agency (EPA) named this metal the ‘King of poisons”. This metal is a Group A carcinogen, and even small amounts may cause injuries to all living organisms (Kalia & Khambholja, 2023). The recommended concentration of As in the lithosphere is 1.8–2.0 mg kg^−1^; concentrations above 20 mg kg^−1^ can promote plant toxicity (Piracha, Ashraf & Niaz, 2019). It is a non-essential toxic metal to plants, hampering the synthesis of photosynthetic pigments, disrupting membrane structure, and causing electrolyte leakage (Nahar et al., 2022). Arsenate can substitute for phosphate in various cellular reactions, causing cellular damage in plants (Sinha et al., 2023). The toxicity of As prompts reactive radicals that are harmful to cell metabolism and the functions of plants (Nabi et al., 2021).

As a consequence of agricultural land contamination with As, it would be of greater interest to find the impact of As on sunflower development (Piracha et al., 2022). *H. annuus,* a stress tolerant crop, belonging to the Asteraceae family, native to North America, is known as “Camp flower”. It is an annual heliotropic plant with straight stems, broader leaves, and coarse hairs. Due to its short growth period of 90-110 days, it can be grown twice a year. The sunflower crop is a major source of oilseeds (Lagiso, Singh & Weyessa, 2021). It is the 4th main oilseed crop and provides a considerable amount of oil content (40–50%) and proteins (17–20%) (Haj Sghaier et al., 2023). As a part of the human diet, planting of this oilseed crop is increasing (Adeleke & Babalola, 2020).

Plants utilize different mechanisms to combat metal stress, including the activation of defense systems and numerous signaling pathways (Noor et al., 2022). Experimenters use various seed treatment methods, such as hydro-priming, osmo-priming, nano-priming, and vitamin priming, to boost the germination percentage and emergence time (Al-Badri, Rasheed & Ahmed, 2021). Priming is an easy approach to combat abiotic factors, such as salinity, drought, and metal stress, hindering the plants growth. Seed priming is a pre-sowing method that enhances the speed of seed germination by breaking seed dormancy (Prajapati et al., 2025). Seed priming promotes activation of metabolic processes during pre-germination stages and allows faster radical growth. The seed priming technique has been widely used in agriculture, especially in response to abiotic stress conditions (Arun et al., 2022).

Seed priming using the B family of vitamins is beneficial for upregulating the antioxidant system. They are important for lowering the singlet oxygen and boosting the plants defensive system. Vitamin-priming is an effective approach to promote plant growth against abiotic stresses *via* seed stress memory. Priming has also been beneficialfor alleviating salinity stress by regulation of salt-response genes along with enhanced membrane stability (Oueslati et al., 2025). Riboflavin (Rib), a water-soluble vitamin and an antioxidant, has two derivatives: Flavin mononucleotide and flavin adenine dinucleotide. Both are active groups in many flavoproteins and coenzymes, essential for various metabolic pathways (Jiadkong et al., 2024).

Rib is indispensable for plant growth and development. It protects the plant by catalyzing various functional and biosynthetic pathways (Aly, Gharib & Ahmed, 2024). This micronutrient plays an important role in stabilizing the health of plants and animals. It gives considerable energy, boosts basal immunity, and provides resistance against infections (Lee et al., 2023). Rib is an efficient vitamin that activates a plant defense system (Mahmoud et al., 2020). It plays an important role in the assembly and signaling of H_2_O_2,_promoting plant immunity against disease and stress management (Vidhyasekaran, 2020). Riboflavin also enhances the regulation of glutathione S-transferase, a detoxification enzyme that scavenges reactive oxygen species (ROS) (Olfat, Ashoori & Saedisomeolia, 2022).

To our knowledge, very few studies have reported the potential of seed priming with Rib to alleviate abiotic metal stress. However, no previous studies were done regarding the ability of Rib to diminish As stress in *H. annuus*. Therefore, we conducted this study to evaluate the potential role of Rib as a priming agent for mitigating As stress in sunflower plants. We hypothesized that Rib priming promotes plant growth by regulating antioxidant response and decreases As toxicity in sunflower plants. The study was designed to understand what role Rib plays in plant growth and development with and without As stress. It was also investigated whether Rib-primed seeds produced different plant growth responses compared with hydroprimed seeds.

## Methodology

### Pot experiment layout

The pot experiment (Fig. 1) was done once in a Complete Randomized Design (CRD). It consisted of four treatments: T0 (Control: Hydro primed seeds), T1 (Priming + no stress), T2 (Stress + no priming), and T3 (Stress + Priming). Each treatment was replicated thrice.

**Figure 1:**
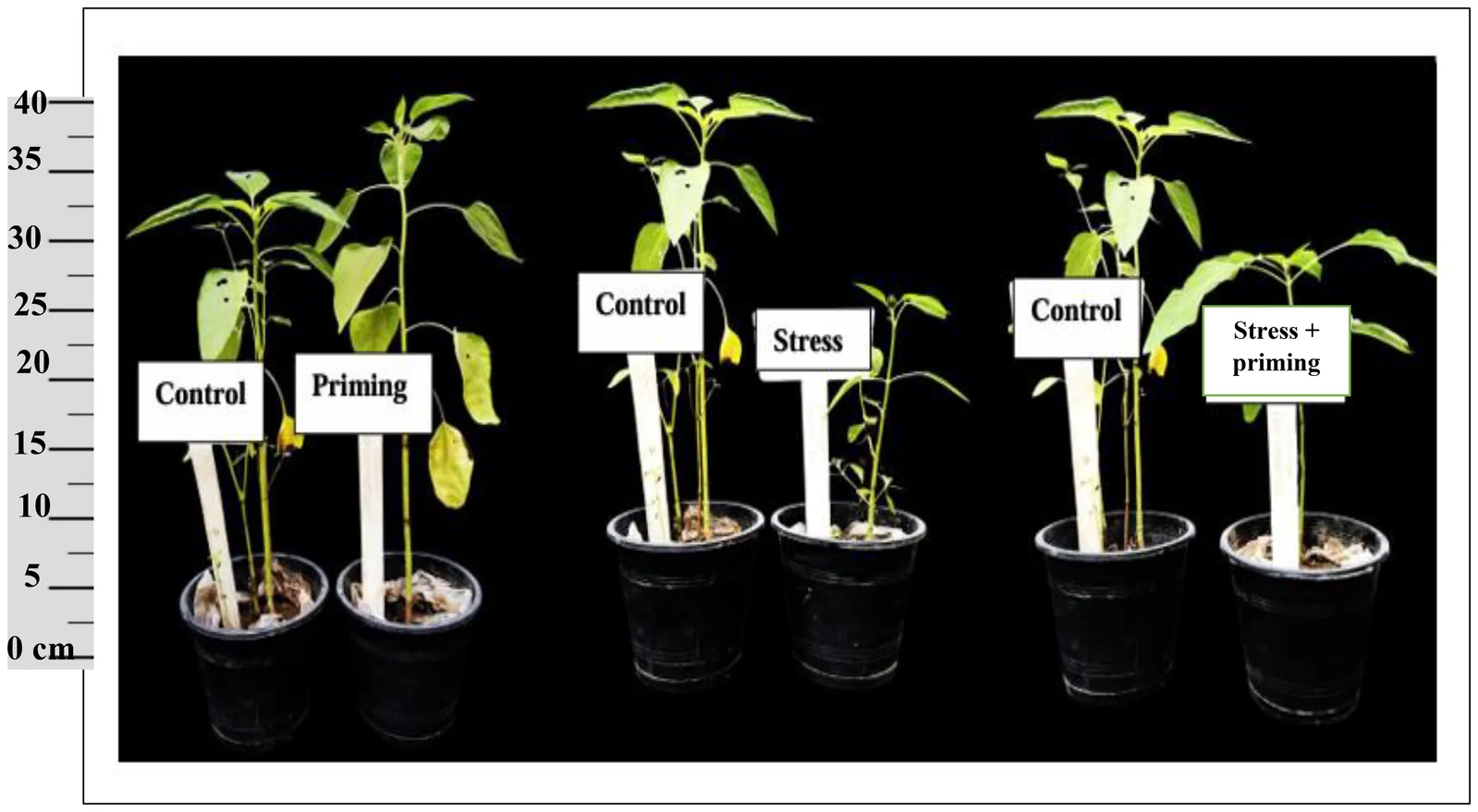
Pictorial presentation of comparing plant growth under control, priming, stress, and stress + priming.

### Seed priming treatment

Seeds of *H. annuus* cultivar ORISUN-751 were purchased from Roshan Seeds Center (Lahore, Pakistan). The seeds were disinfected with a 15% diluted sodium hypochlorite (NaOCl) solution for 5 min and rinsed three times with distilled water. For the screening test, firstly, in a glass jar, sterilized seeds were immersed in 100 ml of Rib solutions of 25, 50, and 75 ppm and incubated for 18 h in the dark at 25 °C. For hydropriming, seeds were immersed in distilled water for 24 h. The variation in the time of soaking was due to the different nature of the priming agents. Hydro-priming includes water uptake, and a more extended period (24 h) is usually necessary to enable enough imbibition. While Rib treatment is associated with exposure to riboflavin, a bioactive compound that speeds up physiological activation. Hence, it was given a short period (18 h) without causing possible overstimulation.

For 25, 50, and 75 ppm solutions of Rib, it was prepared by dissolving 25, 50, and 75 mg of granular Rib in one liter of water, respectively. Then on a petri plate, ten healthy seeds were used for all treatments, and hydro-primed seeds were used as the control. Three replicates per plate were selected for germination parameters to screen the effective Rib concentrations. Results showed that 50 ppm performed better among others (File S1). After performing the screening test, the optimal concentration for the rest of the experiments was taken as 50 ppm of Rib.

### Soil spiking and seed sowing

The contaminated soil was prepared by thoroughly mixing it with a 10 ppm As solution. The concentration was taken from studies of Pant, Tripathi & Dwivedi (2011) and Singh et al. (2021). For 20 days, the soil was placed in the shade, then pots were filled with 1 kg of contaminated soil. Primed and hydroprimed seeds were sown and irrigated with distilled water along with a 50% Hoagland solution at one-week intervals until harvest (90 days). The Hoagland solution’s composition was as follows *i.e.,* Fe-EDTA 40 μM, H_3_BO_3_ 25 μM, MnCl_2_.4H_2_O, 2 μM, CuSO_4_.5H_2_O, 0.5 μM, KCl 50 μM, (NH_4_)_6_Mo_7_O_24_.4H_2_O, 0.075 μM, KNO_3_, 2 mM, NH_4_NO_3_, 0.5 mM, MgSO_4_.7H_2_O, 0.5 mM, KH_2_PO_4_, 0.25 mM. At the end of the experiment, the plants were harvested and separated into roots and shoots. Plant shoot length, fresh weight, and dry weight were determined (Fig. 2).

**Figure 2:**
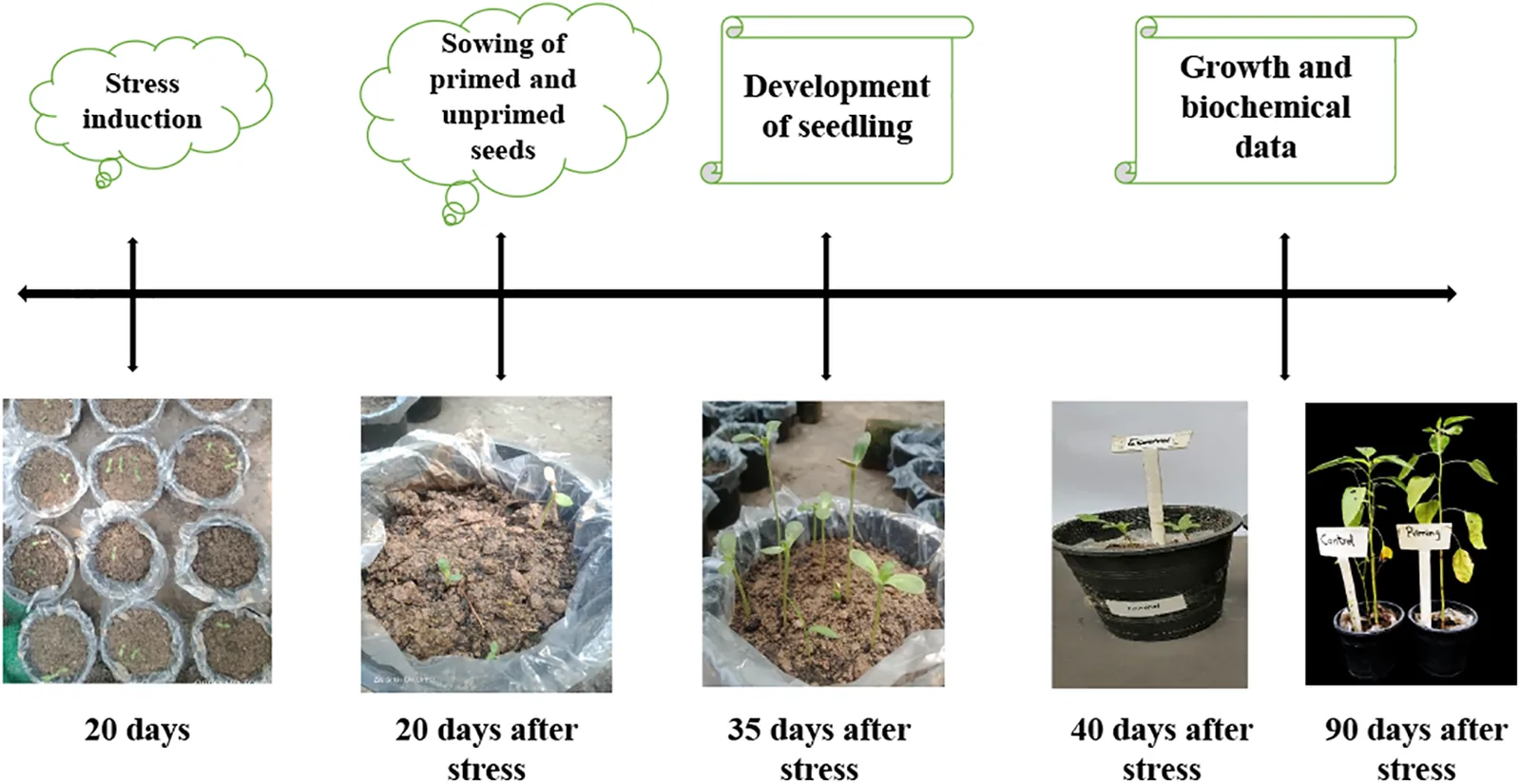
Plan of stress induction and experiment execution.

### Experimental soil characteristics

We collected the soil used in pots from a site near the experimental area at a depth of 0–0.5 m. The physicochemical attributes of the soil were assessed by adopting the standard methods proposed by Rhoades (1982). The soil had a clay loam texture with a pH of 7.6–8.0 and a water content between 0.37% and 0.90%. EC of soil is between 1.6 and 4.7 mS cm^−1^, organic matter is between 0.50% and 0.81%, and phosphorus is between 0.3 and 0.8 mg kg^−1^ dry weight of soil. Potassium is between 12 and 159 mg kg^−1^, calcium is between 0.15 and 0.20 mg g^−1^, magnesium is between 0.29 and 0.30 mg g^−1^, chloride is between 0.43 and 1.63 mg g^−1^, bicarbonates are between 2.01 and 2.22 mg g^−1^, and phosphate is 1.76–2.34 mg g^−1^.

### Estimation of electrolyte leakage and membrane stability index percentage

For electrolyte leakage (EL) and membrane stability index (MSI) examinations, we placed a leaf (0.1 g) into a test tube with 10 mL of distilled water (Sairam, 1994). First, we heated the test tubes in a water bath at 40 °C for 30 min. Next, we removed the test tubes and let them cool for a bit. We measured the conductivity (C1) of all the samples using a probe. Then, we heated the samples in the water bath at 100 °C for 10 min and recorded the conductivity (C2) value. To assess the membrane stability index, we used the formula as follows:

\begin{eqnarray*}MSI~(\%)= \left[ 1- \frac{C1}{C2} \right] \times 100 \end{eqnarray*}

EL was calculated according to the method and formula proposed by Sullivan & Ross (1979)

\begin{eqnarray*}EL~(\%)= \frac{C1}{C2} \times 100. \end{eqnarray*}

### Estimation of photosynthetic content

To estimate photosynthetic content, 0.5 g of fresh sunflower leaves was taken and mashed using a pestle and mortar. Followed by mashing, the sample was put in a Falcon tube containing 10 mL of 80% acetone. The tubes, enclosed with aluminum foil, were placed in the dark for 48 h. Spectrophotometric (Lamda-35-UV–VIS Spectrometer) value of Chl *a*, Chl *b*, total chlorophyll, and carotenoid content determined by observing OD at 663, 645, and 470 nm. The following equations determined each sample’s chlorophyll content (Lichtenthaler, 1987) and carotenoid content (Lichtenthaler & Wellburn, 1983).

\begin{eqnarray*}Chlorphyll~a~content~(mg~{g}^{-1})& =12.21 \left( A663 \right) -2.81(A645) \end{eqnarray*}

\begin{eqnarray*}Chlorphyll~b~content~(mg~{g}^{-1})& =20.13 \left( A645 \right) -5.03(A663) \end{eqnarray*}

\begin{eqnarray*}Total~chlorophyll~(mg~{g}^{-1})& =20.2 \left( A645 \right) +8.02(A663) \end{eqnarray*}

\begin{eqnarray*}Carotenoid~content~(mg~{g}^{-1})& = \frac{1000A470-3.27 \left( chl~a \right) -104(chl~b)}{227} \end{eqnarray*}

### Estimation of malondialdehyde content

The malondialdehyde (MDA) content was estimated from the quantity of reactive substances, using 2-thiobarbituric acid (Senthilkumar, Amaresan & Sankaranarayanan, 2020). As per the standard method, we crushed 0.1 g of the leaf sample in a clean pestle and mortar in the presence of two mL of 0.1% trichloroacetic acid (TCA). The extract was later centrifuged for 15 min. The supernatant (one mL) was mixed with four mL of 0.5% TBA and one mL of TCA and heated in a water bath for 30 min at 95 °C. The reaction mixture was cooled, and OD was obtained at 440 nm, 532 nm, and 600 nm.

### Determination of gas exchange parameters

Using plant leaves, we measured gaseous exchange parameters with an infrared gas analyzer (IRGA) equipped with a stable leaf chamber (LI-COR Inc., Lincoln, NE, USA). We scheduled measurements of transpiration rate (E), stomatal conductance (gs), and photosynthetic rate (A) between 9:00 and 10:00 am. A mature leaf was gently placed inside the chamber, and readings were noted for each replicate to ensure accuracy. The temperature inside the leaf chamber (Tch) was carefully adjusted between 32–36 °C. Additionally, the atmospheric pressure was consistently adjusted to 986 millibars to create optimal conditions.

### Assessment of protein content (mg g^−1^ FW)

Protein assessment was achieved by the Bradford method (Bradford, 1976). A leaf sample of 0.1 g was flattened along with one mL of sodium phosphate buffer (pH 7.5) in a clean mortar. The mixture was centrifuged for 10 min (3,000 rpm). Supernatant (0.1 mL) was mixed with 0.9 mL of distilled water in a tube. Bradford Reagent (five mL) was later added to the test tubes and incubated at room temperature for 30 min. The OD of the solution was taken at 595 nm.

### Determination of antioxidant enzymes

To assess the activity of the antioxidant enzyme, a fresh leaf sample (0.5 g) was taken and homogenized in phosphate buffer (five mL). The solution was centrifuged at 13,000 g for 20 min. The supernatant was separated and utilized to prepare enzyme assays. A reaction sample solution was prepared in titanium reagent for the catalase activity, and OD was measured at 410 nm after 30 min using a spectrophotometer (Lamda-35-UV–VIS Spectrometer; Perkin Elmer) (Zhang et al., 2017). Peroxidase activity was determined using the method of Jiang et al. (2018), measuring absorbance at 485 nm for 1 min. For APX activity, the absorbance of the reaction solution was obtained at 290 nm for 2 min.

### Mass spectrometry-gas chromatography

The phytocompounds linked to heavy metal stress tolerance were identified by mass spectrometry-gas chromatography (GC-MS). For this purpose, before the flowering stage, a leaf sample from the plant treated with the applied treatments was collected using the protocol proposed by Nam et al. (2016). For metabolomics profiling of leaf tissues, one g of freshly plucked leaf sample was weighed, mashed into a fine powder in a chilled, clean mortar using liquid nitrogen. Followed by five mL of 100% methanol solution to make a homogenate mixture. The mixture was placed in Eppendorf tubes and then kept in the refrigerator. After 24 h, At room temperature, the reaction sample was centrifuged at 13,000 rpm for 10 min, and 20 μL of the supernatant was collected. Later, filtered the supernatant using microfilters (0.2 µm), and transferred it into the sample vials. The sample vials were sent to the Textile Testing Lab (ISO: 17025) to detect various metabolites. Analysis of GC–MS used the instrument (Agilent Technologies Model: 7890B, 5977A), fitted with a DB-5MS capillary column. GC conditions were included: carrier gas He, one μL injection volume, 275 °C injector temperature, and a 50–500 m/z mass range. Data were processed using MzMine V. 2.53 software (Pluskal et al., 2010).

### Statistical analysis

We designed a pot experimental trial, comprising four treatments, each replicated thrice. Statistical analysis was performed to obtain the mean value and its standard error of the mean with a significance level P = 0.05. To confirm the variation between the groups, analysis of variance (ANOVA) was performed. A *post-hoc* analysis of Duncan’s multiple range test (DMRT) was conducted to determine whether there were any significant differences among the treatments. The statistical analyses were performed using IBM SPSS Statistics 20 software. A graphical display of the data was created using OriginPro 2024 (https://www.originlab.com/2024).

## Results

### Morphological growth traits

The results revealed that T2 (stress + no priming) reduced plant height to 22.33 cm (40.5%) in contrast to the primed seeds. The T1 (priming + no stress) significantly augmented plant height to 37.54 cm compared with other treatments. Plant fresh (15.7 g) and dry weight (2.43 g) also showed enhancement when exposed to T1. However, the differences in plant dry weight remained negligible among all treatments except T1 (Figs. 3A–3C).

**Figure 3:**
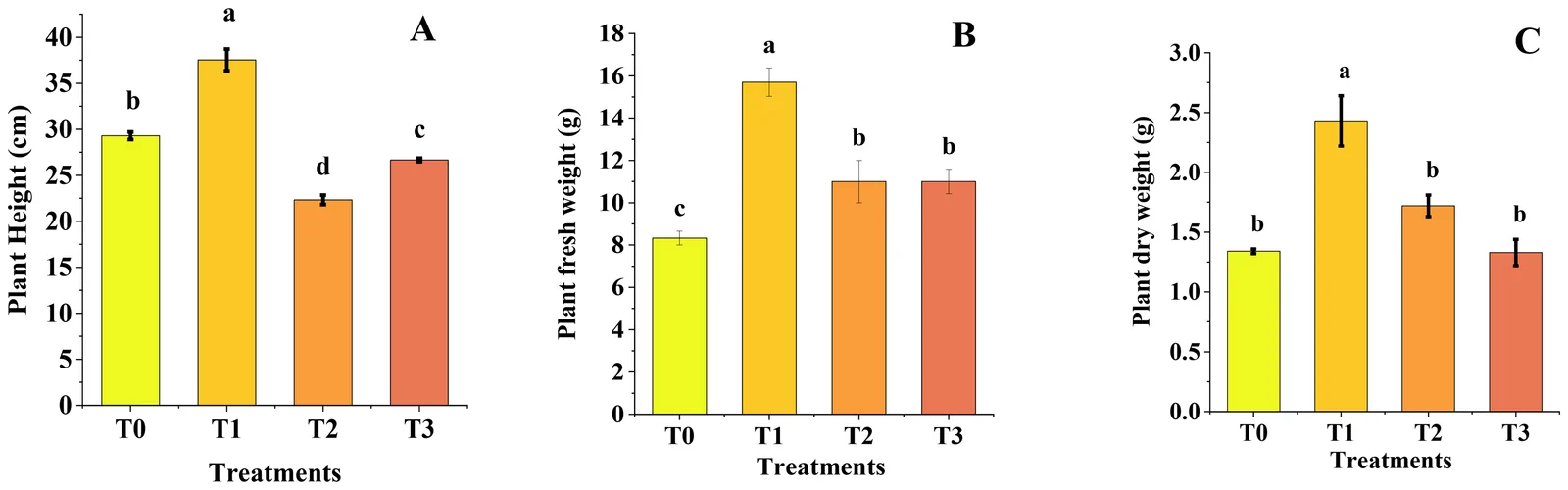
The effect of riboflavin on the growth parameter plant height (A), plant fresh weight (B), and plant dry weight (C) in *H. annuus* under As stress. Error bars in graphs present standard error, and alphabets show heterogeneous groups obtained from *post-hoc* analysis of Duncan’s multiple range test (DMRT)”. Values represent mean ± SEM (*n* = 3). Different letters above the bars indicate significant difference among treatments (*p* ≤ 0.05). T0 (control), T1 (Priming+ no stress), T2 (Stress+ no priming), T3 (Stress+ Priming).

### EL, MDA, and MSI

Relative to T0, T2 increased electrolyte leakage from 23.45% to 54.43% (132.11%) and MDA from 26.12 to 62.25 nmol mL^−1^ (138.32%), while reducing MSI from 76.55% to 45.57% (40.47%). Relative to T2, T3 reduced electrolyte leakage to 44.19% (18.81%) and MDA to 51.03 nmol mL^−1^ (18.02%), while increasing MSI to 55.80% (22.45%). T1 exhibited the lowest electrolyte leakage (18.41%) and highest MSI (81.59%) (Table 1).

**Table 1:** Effect of seed priming on MDA, electrolyte leakage, and membrane stability index (MSI) in response to the applied treatments. Means of three replicates and standard error of mean followed by letters displaying significant difference with *p* ≤ 0.05.

**Treatments**	**MDA** **(nmol mL** ^−1^ **)**	**Electrolyte leakage (%)**	**MSI (%)**
**T0**	26.12 ± 1.44 c	23.45 ± 1.68 c	76.55 ± 1.68 b
**T1**	33.62 ± 2.72 c	18.41 ± 1.32 d	81.59 ± 1.32 a
**T2**	62.25 ± 3.81 a	54.43 ± 1.54 a	45.57 ± 1.54 d
**T3**	51.03 ± 2.76 b	44.19 ± 0.38 b	55.80 ± 0.38 c

**Notes.**

Where T0 = Control, T1 = Priming + no stress, T2 = stress + no priming, and T3 = Stress + Priming, data presents Means ± standard error and alphabets present means comparison test.

### Green pigment content and gas exchange attributes of plant leaves of *H. annuus*

The results indicated noticeable changes in pigment synthesis between the treatments. Compared with other treatments, we observed a significantly increased level of total chlorophyll (13.17 mg g^−1^), followed by T3 (11.18 mg g^−1^). However, plants grown under stress (T2) displayed a mean value of 8.87 mg g^−1^, a 22.30% reduction in total chlorophyll content in comparison to T0. We observed increased gas exchange parameters of *H. annuus* plants that underwent T1 (priming + no stress). The T1 treatment showed increased photosynthetic rate to 9.92-µmol m^−2^s^−1^ up to 92.29% and stomatal conductance to 0.07 mol m^−2^s^−1^ (90.00%) compared to the control. While T2 (stress + no priming) administration reduced the photosynthetic activity, by reducing the photosynthetic rate to 1.73 µmol m^−2^s^−1^ (−66.52%), stomatal conductance to 0.02 mol m^−2^s^−1^ (−54.49%), and transpiration rate to 0.13 mol m^−2^s^−1^ (−87.52%) in comparison to untreated plants (Figs. 4B–4D).

**Figure 4:**
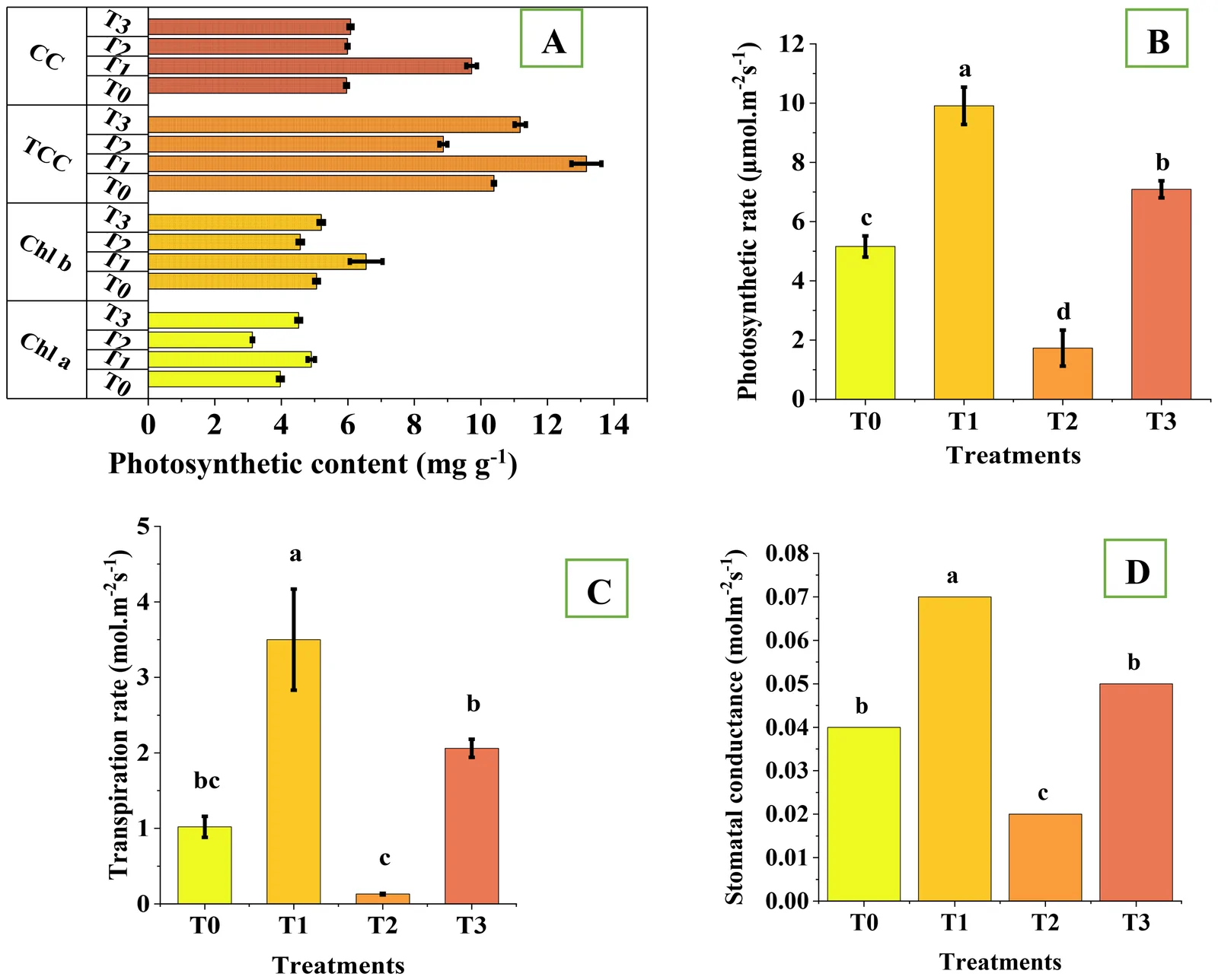
The impact of riboflavin on photosynthetic content (Chl a, Chl b, CC; carotenoid content, TCC; total chlorophyll), photosynthetic rate, transpiration rate, and stomatal conductance *H. annuus* under As stress. Error bars in graphs present standard error and alphabets show heterogeneous groups obtained from *post-hoc* analysis of Duncan’s multiple range test (DMRT)”. CC and TCC stand for carotenoid contents and total chlorophyll contents respectively. Where T0 = Control, T1 = Priming + no stress, T2 = stress + no priming, and T3 = Stress + Priming, data presents Means ± standard error and alphabets present means comparison test.

### Protein content and antioxidant enzyme activity

We observed maximum protein content, (1.12 mg g^−1^ FW) in plants grown under T2. In comparison, T1 showed a reduction in protein content to 0.17 Umg-1 FW, followed by T3. The results showed that T1 increased the activity of POD, CAT, and APX, with CAT increasing to 1.52 U mg^−1^ (3-fold), POD to 3.04 U mg^−1^ (2-fold), and APX to 16.17 µmol g^−1^ (6-fold) compared to the untreated plants (T0). In plants, administered T3, POD and CAT activities were significantly higher than those of T0 and T2. The T3 (stress + priming) increased the CAT activity to 0.63 U mg^−1^compared to T0 and T2, while POD increased by 16.16% compared to T0 (control). However, APX activity remained non-significant in T0, T1, and T3 (Figs. 5A–5D)

**Figure 5:**
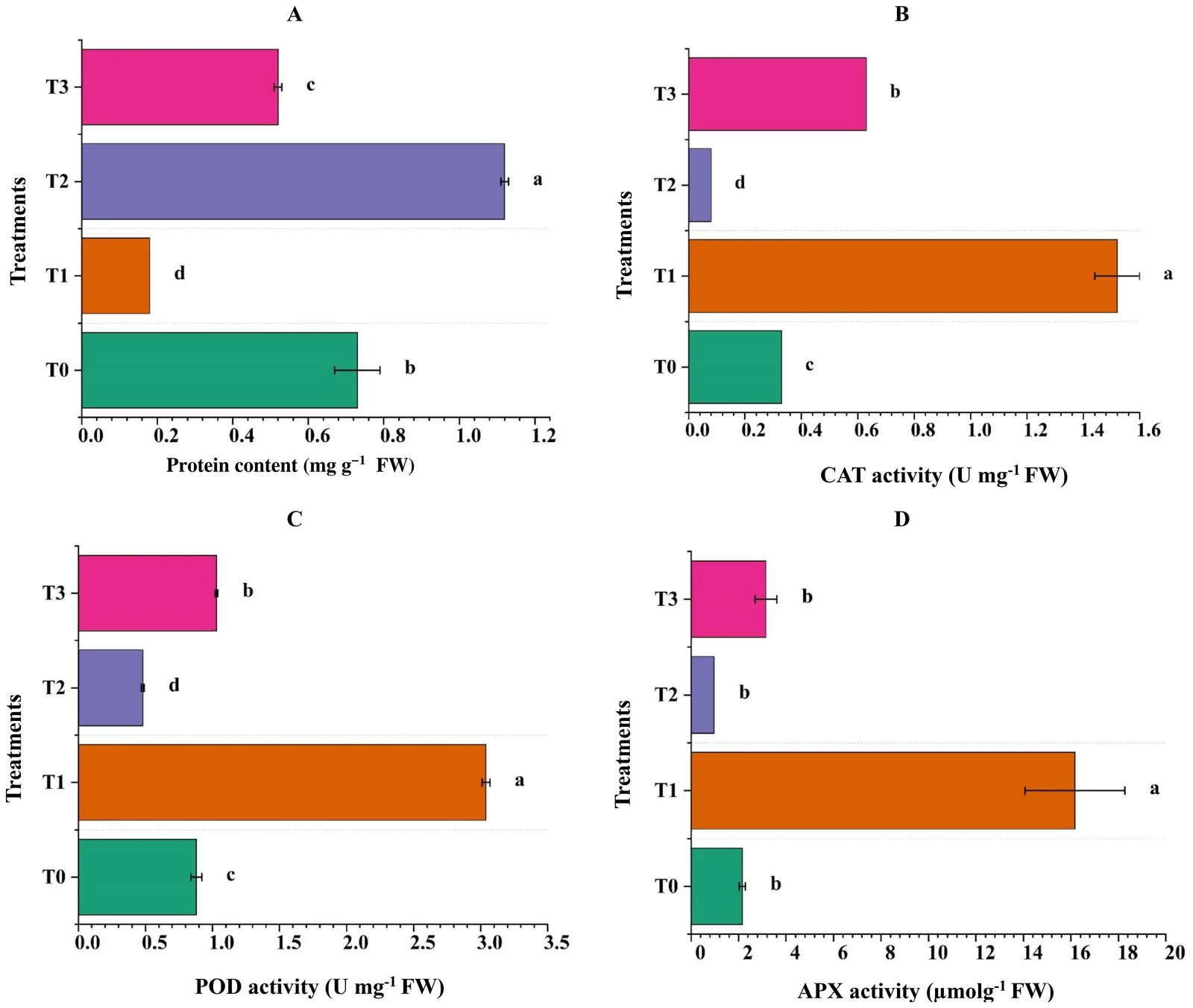
The effect of riboflavin on protein content and antioxidants (CAT; catalase activity, POD; peroxidase activity, APX; ascorbate peroxidase activity) in *H. annuus* under As stress. Error bars in graphs present standard error and alphabets show heterogeneous groups obtained from *post-hoc* analysis of Duncan’s multiple range test (DMRT)”. Values represent mean ±SEM (*n* = 3). Different letters above the bars indicate significant difference among treatments (*p* ≤ 0.05). T0 (control), T1 (Priming+ no stress), T2 (Stress+ no priming), T3 (Stress+ Priming).

### Metabolites profiling (GC-MS)

A list of metabolites was detected in *H. annuus* grown under different experimental conditions (File S1). These included various classes of compounds as shown in Fig. 6. The most abundant phytocompounds detected in the control plants were triacylglycerol (TAG) with Retention Time (RT) 18.18 min and base peak area (BPA) 4.41%, benzoquinone (BQ) with RT:18.31 min and BPA: 1.55%, glycolate (GA) with RT:18.16 and BPA: 1.58%, and L-cysteine (L-Cys). The compounds present in the highest amount in the T3 group were Aminopropiononitrile (APN) having RT: 18.67 min and BPA: 2.94%, Choline (Cho) showing BPA: 2.24% at RT: 18.32, D-Glucono-1,5-lactone (GDL) displaying the abundance at RT: 9.54 min with BPA: 2.8%, GA, Isoprene (IP), and Phenylacetaldehyde (PAA) with RT: 18.32 min and BPA: 2.41%. The phytocompounds noted under T2 (stress + no priming) were Acrolein (Acr) and IP.

**Figure 6:**
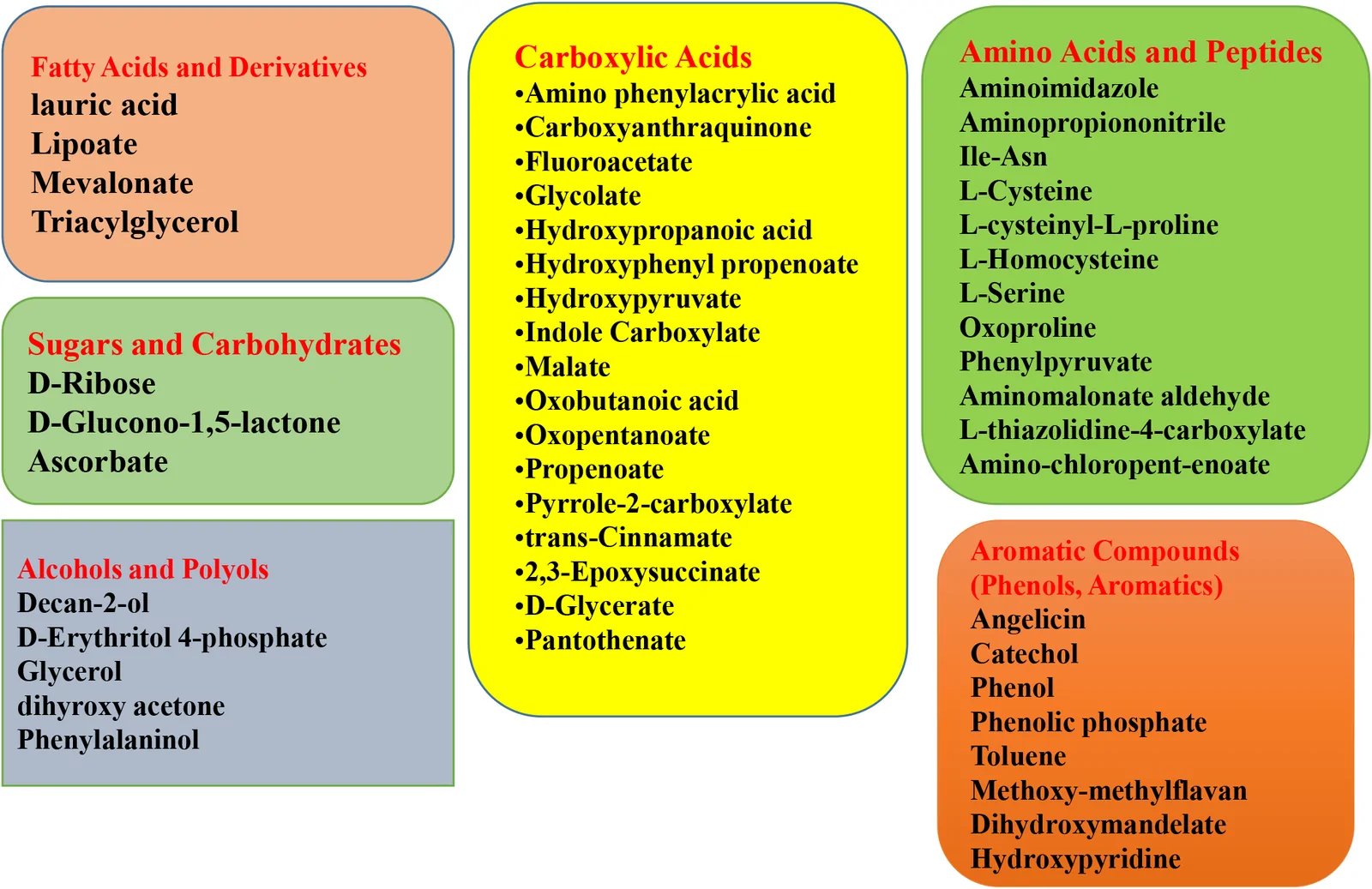
Metabolic pathway model of alterations in the metabolome of *H. annuus* grown under As-stress caused by riboflavin seed priming.

The results indicated that the maximum percentage of GDL (2.80%) was in Rib-treated plants grown under stress (Table 2). The heat map visually displays the relative abundance of different identified phytocompounds during GC-MS analysis of *H. annuus* (Fig. 7A–Fig. 7B). The T3/T0 and T1/T0 treatments showed the upregulation of compounds, indicated by widespread dark areas. The chromatographic analysis further revealed these dynamic changes in metabolites (Figs. 8A–8D). These distinct profiles illustrate the potential impact of each treatment on sunflower metabolism.

**Table 2:** Metabolic compounds percent composition detected in *H. annuus* samples in response to the applied treatments T0 (Control), T1 (Priming + no stress), T2 (Stress + no priming), T3 (Stress + Priming).

**Retention time (min)**	**Metabolites**	**Normalized base peak area %**			
		**T0**	**T1**	**T2**	**T3**
9.54	D-Glucono-1,5-lactone	1.96	1.35	0.2	2.8
17.98	Hydroxyphenyl propenoate	0.05	0.43	0.04	0.04
18.31	Benzoquinone	1.55	1.58	0.68	0.74
18.67	Aminopropiononitrile	1.41	1.78	2.17	2.94
18.67	Acrolein	2.36	3.83	4.27	3.68
18.32	Choline	1.96	1.43	1.73	2.24
18.18	D-Glycerate	0.7	1.07	0.25	1.27
18.16	Glycolate	1.58	0.35	1.2	1.48
19.06	Isoprene	2.11	3.24	3.48	3.49
18.42	L-Cysteine	0.73	0.7	0.36	0.04
18.85	L-cysteinyl-L-proline	0.01	0.27	0.12	0.01
18.09	Phenol	2.48	2.28	3.07	3.19
18.21	L-Homocysteine	0.56	0.02	0.13	0.15
18.32	Phenylacetaldehyde	1.2	1.24	1.49	2.41
18.18	Triacylglycerol	4.41	2.85	2.76	1.07
18.23	L-Serine	0.07	0.05	0.05	0.08

**Figure 7:**
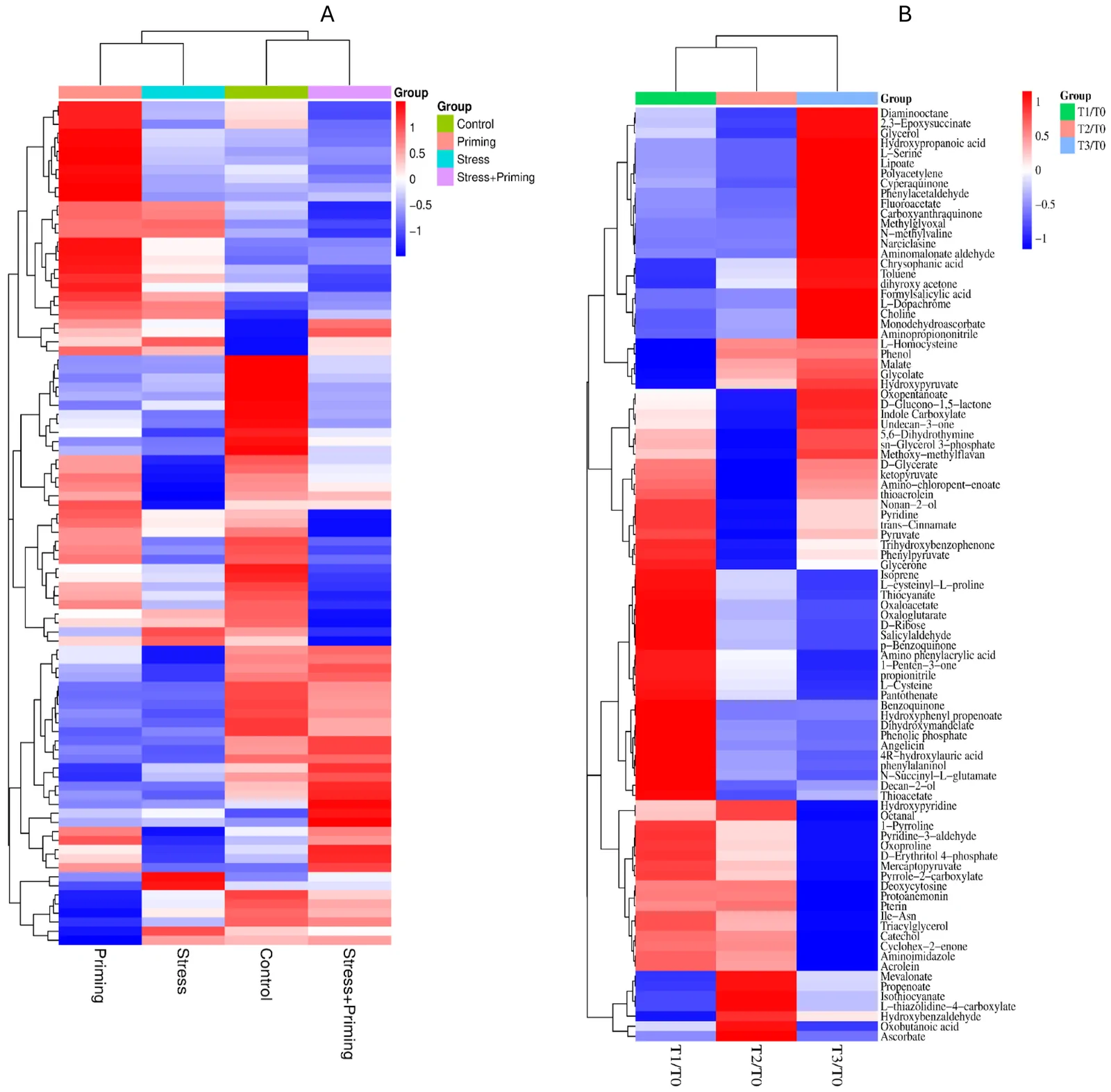
Heatmap of the relative abundance of metabolites in each of the four treatments: Control (T0), Priming + no stress (T1), Stress + no priming (T2), and Stress + Priming (T3).

**Figure 8:**
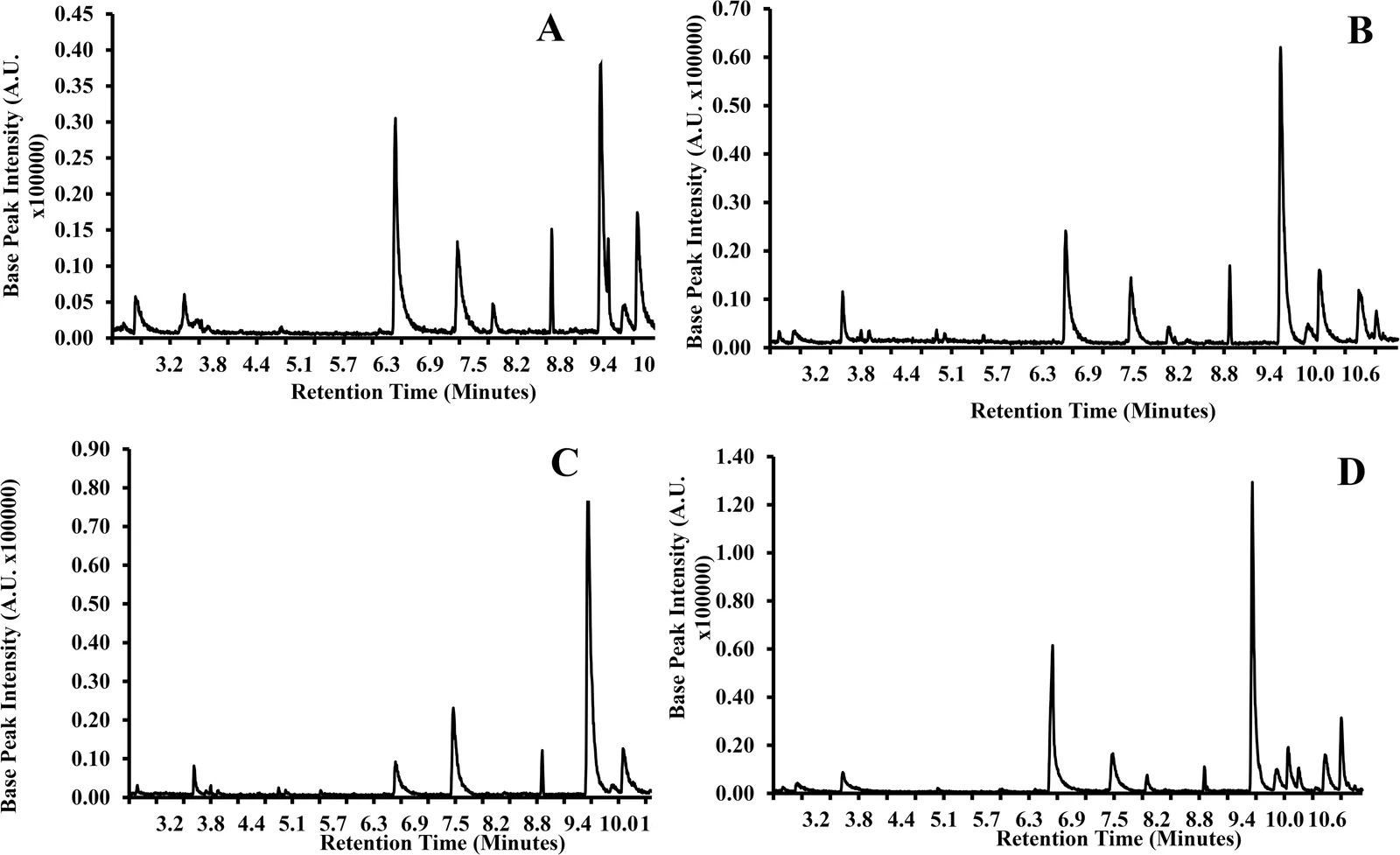
Chromatograms (TIC, scan mode) in the non-targeted GC-MS analysis to study a mix of different metabolites from *H. annuus* in response to Control (A), Priming + no stress (B), Stress + no priming (C), and Stress + Priming (D).

### Pearson correlation between traits of riboflavin-primed *H. annuus* planted in soil contaminated with As

To understand the reciprocal effect among growth attributes of *H. annuus* plants, a simple correlation coefficient was calculated. It was observed that plant height showed positive correlation with carotenoid content, with *p* value equal to 0.58, total chlorophyll (*r* = 0.46), chlorophyll a (*r* = 0.30), and chlorophyll b (*r* = 0.58) according to the Pearson correlation. The gas exchange parameters, photosynthetic rate (*r* = 0.73), transpiration rate (*r* = 0.67), and stomatal conductance (*r* = 0.75) all showed positive correlations with the membrane stability. The MDA content showed a relatively negative correlation with total chlorophyll (*r* = −0.56), gas exchange attributes, and protein content. The antioxidant enzyme activities CAT, POD, and APX positively correlated with each other (Fig. 9).

**Figure 9:**
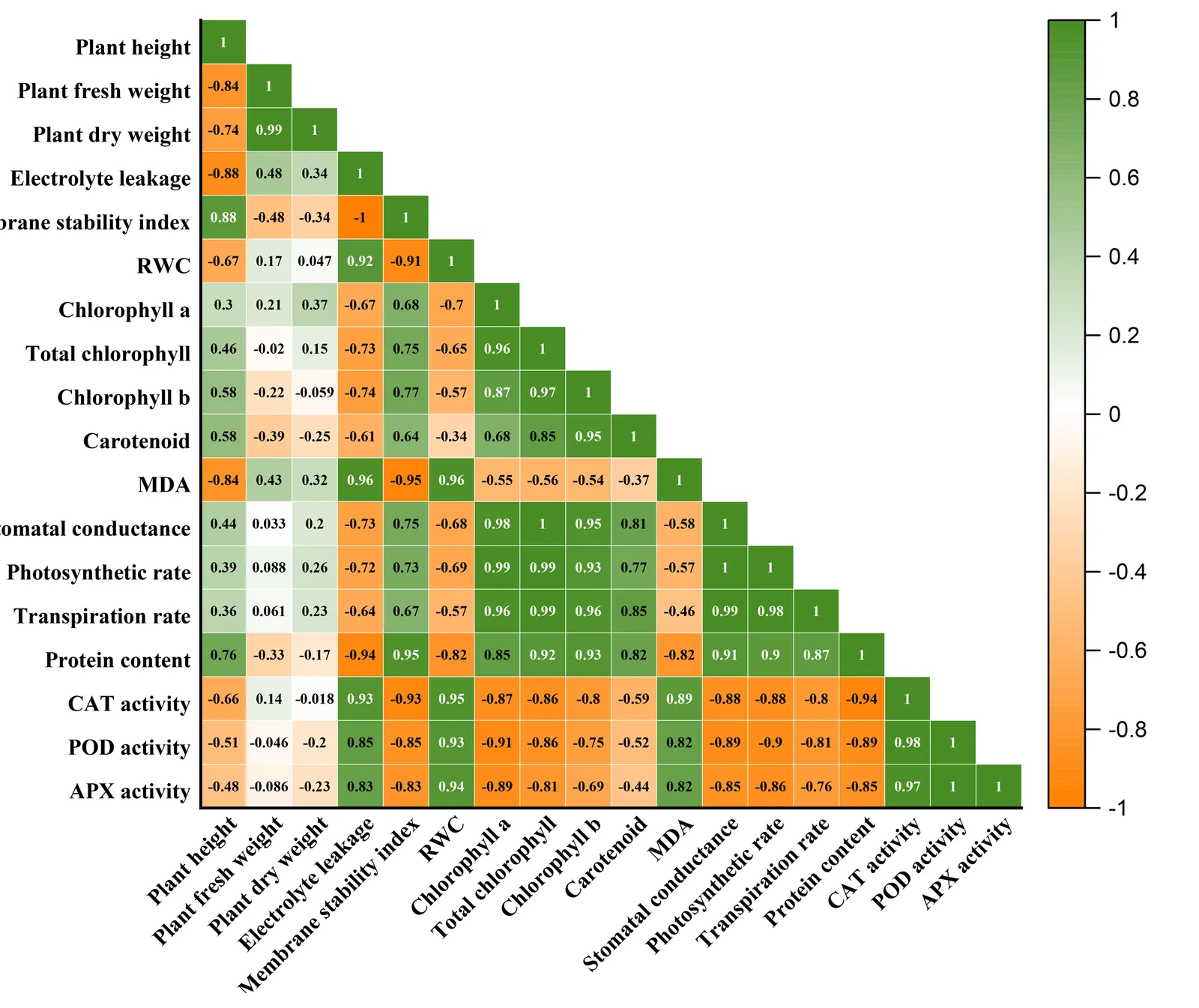
Correlation coefficients among the growth attributes of *H. annuus* grown in As-contaminated soil.

### Principal component analysis

The growth of *H. annuus* on soil amended with As, and primed with Rib, showed a correlation among the traits in response to the applied treatments. This relationship shows the loading plots obtained from principal component analysis (Fig. 10). It illustrates two main components, PCA 1 and PCA 2, which account for over 91.82% of the entire dataset. PCA 1 accounted for 71.72% of the data, while PCA 2 accounted for 19.08%. The parameters, including plant growth attributes, plant fresh weight, plant dry weight, and physiological attributes, electrolyte leakage, and MDA content, showed a positive correlation because of T3 (stress + priming). Similarly, the plants subjected to T1 (priming + no stress) displayed a positive relationship among antioxidant enzyme activities.

**Figure 10:**
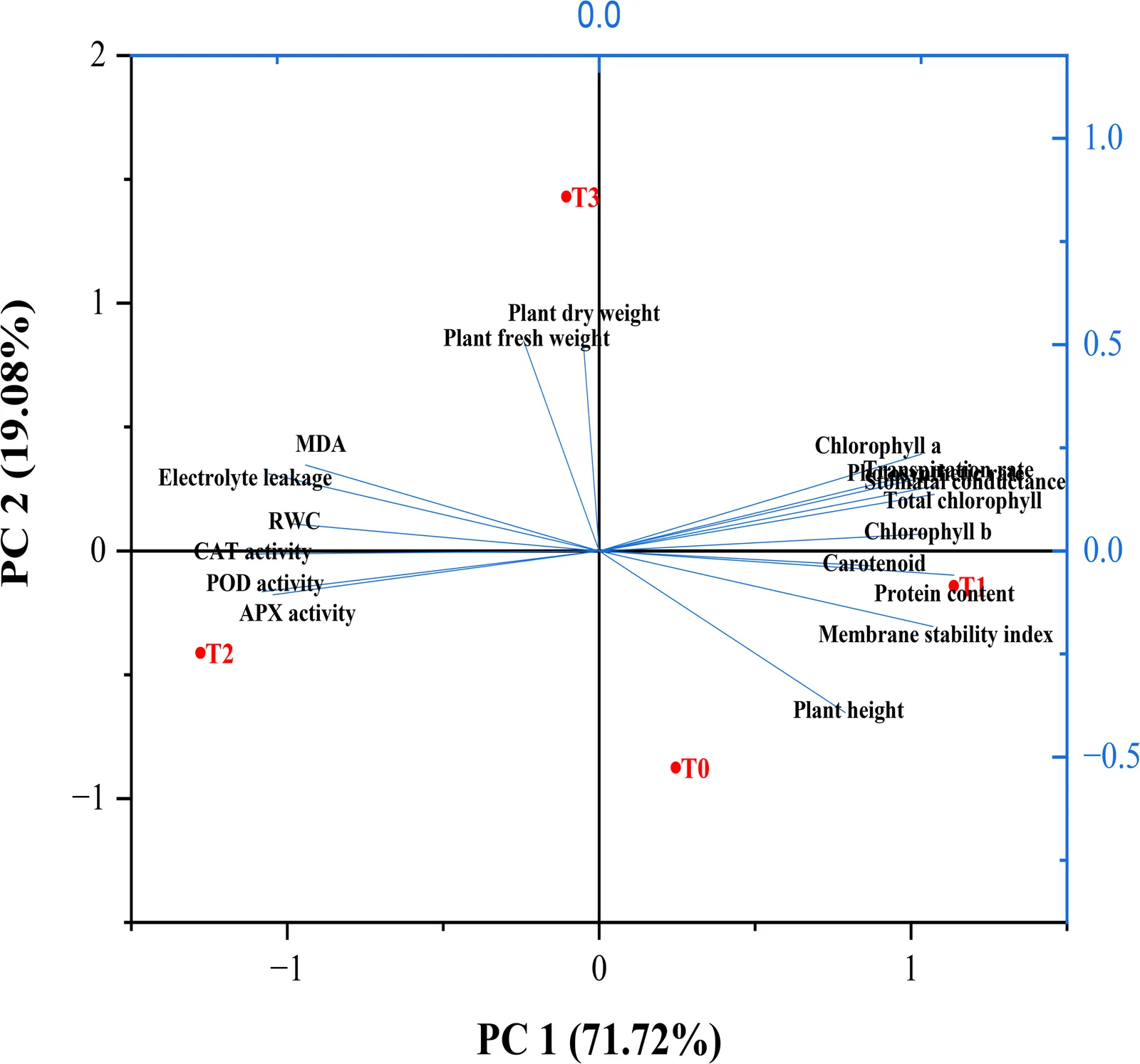
Principal component analysis (PCA).

### Discussion

In sustainable agriculture, soil contamination with heavy metals raises major concerns. These metals hinder plant growth and compromise the plant’s yield by damaging the antioxidant system (Feng et al., 2023). Among the heavy metals, As is of more importance, due to its toxicity in land resources. It affects the physiological and biochemical processes of plants, leading to growth abnormalities (Singh et al., 2021). In this context, we designed a study to alleviate As stress and its effects on growth characteristics through seed priming with Rib. Rib, a growth promoter, was used as a priming agent due to its potential to enhance photosynthetic content, strengthen the antioxidant defense system, improve nutrient uptake, and enhance growth attributes (Deng et al., 2014). The current study revealed that plants subjected to As stress showed a significant decrease in growth parameters. This reduction in plant height and dry weight is ascribable to diminished membrane stability, increased MDA content, and increased electrolyte leakage. Nawaz et al. (2024) reported similar results in *H. annuus* under As stress, with enhanced reactive oxygen species generation, primarily as reflected in MDA content. Plants’ reduced height and dry weight are attributable to the negative impact of As stress on cell division, membrane permeability, leading to leakage of essential nutrients. The reduced growth can also be linked to an interruption in photosynthetic machinery and As’s negative effects on plants’ ground and aerial parts (Ibrahim et al., 2026). Piracha et al. (2022) reported similar findings, in which As stress in *H. annuus* reduced plant growth by increasing electrolyte leakage and reducing photosynthate production. However, priming with Rib results in better plant growth, owing to its role in improving water and mineral nutrient uptake (Ren et al., 2026). Rib has a role in cell division, leading to better germination, inducing vigour in the plant and enhanced nutrient uptake (Tian et al., 2022).

Similarly, Rib is an antioxidant that enhances plant growth under abiotic stress. It helps them absorb minerals crucial for growth (Chen et al., 2017). It also increases photosynthesis by stabilizing membrane integrity against the production of radical species and their harm to the cell. Plants under As stress often show reduced pigment synthesis, mainly due to As’s damaging effects on the chlorophyll machinery. It affects the Calvin cycle and its enzymes by reducing the efficiency of photosystem I and II, and by causing stomatal closure. It also reduces the production of chlorophyll-synthesising enzymes, such as chlorophyllase. Our results are in accordance with Chen et al. (2017) and Garg & Cheema (2021), who found that As stress impeded photosynthetic activity in cucumbers and chickpeas.

Seed priming with Rib helps the plants to produce their pigment synthesis, owing to the role of Rib in stomatal opening and reduction in chlorophyll degradation. Riboflavin improved chlorophyll uptake by reducing oxidative damage. Iftikhar & Perveen (2024) obtained similar results in *Zea mays* L. under abiotic stress. Under abiotic stress, plants undergo numerous metabolic changes (Yang, Tang & Zhao, 2026). We noted that stress impeded the plants’ ability to synthesize chlorophyll. According to our observation, As stress reduced plant gas exchange attributes, attributable to the generation of ROS in response to As stress, which diminishes plant physiological and biological processes. Das & Biswas (2022) also observed similar findings in rice seedlings. The reduced photosynthetic rate is linked to stomatal factors, reduced stomatal density, pore area, and malfunctioning of the reaction center. As a result, plants are unable to synthesize porphobilinogen, a compound essential for chlorophyll synthesis. Patel & Parida (2022) reported similar findings in *Salvadora persica* L. in response to As stress.

In our study, reduced transpiration rate was linked with water loss from the plant’s surface in response to As stress. Anjum et al. (2017) also reported reductions in carbon fixation, stomatal conductance, transpiration rate, and net photosynthetic activity under As and cadmium stress in *Z.mays*. However, we found that seed priming with Rib increased gas exchange activity, ascribable to its role in the production of green pigments, greater carbon fixation, and stomatal conductivity. Abd-Rabbu et al. (2024) reported similar findings, in which Rib application increased gas exchange parameters in marjoram. Our findings demonstrate that Rib priming can reduce As stress in sunflower plants by regulating chlorophyll synthesis and stomatal index, thereby increasing gas exchange parameters (Wu et al., 2024).

According to our results, As promoted protein content while reducing antioxidant activity in sunflowers. A decrease in antioxidants is attributable to the production of free radicals and oxidative stress. These ROS cause damage to membrane stability and uptake of water and minerals (Nahar et al., 2022). Plants undergo oxidative stress, which impairs their defense systems. Our results are consistent with previous studies by Tripathi et al. (2016) and Kashif et al. (2021). Under abiotic stress conditions, plants activate their innate defense mechanisms, which help them manage and survive against the production of reactive oxygen species (Maghsoudi, Arvin & Ashraf, 2020). Our findings showed that seed pre-treatment with Rib increased the production of peroxidase, catalase, and ascorbate peroxidase, allowing the plants to survive in stress conditions (Deng et al., 2014). Aly, Gharib & Ahmed (2024) and Zha et al. (2022) reported similar conclusions on rosemary and apple, respectively.

Our study found that abiotic stress reduced amino acid and organic acid production (Dewan et al., 2026), thereby decreasing phenyl pyruvate and pyruvate levels. These metabolites are crucial for glycolysis and the tricarboxylic acid cycle. Phenyl pyruvate is a keto acid and an amino acid precursor, playing a key role in many plants’ metabolic pathways. Pyruvate is a central molecule in cellular respiration (Galili, Amir & Fernie, 2016). The negative impact of stress likely caused this, disrupting carbon flow, amino acid production, enzyme activities, and energy generation (Trovato et al., 2021). Stressed plants also produced more acrolein, a reactive carbonyl species and singlet oxygen molecule, indicating their stress response (Biswas & Mano, 2021). However, plants treated with seed priming during stress showed slight changes in these compounds, mainly due to Rib’s role in stabilizing primary metabolic pathways and mitigating As stress (Jiadkong et al., 2024).

Our study revealed that decreased membranous stability due to abiotic stress was attributable to reduced antioxidant defense systems. In our study, the priming treatment increased the proportion of specific phenolic and fatty acid compounds that help plants protect themselves against abiotic stresses (Nahar et al., 2022). Rib’s role as a precursor for various enzyme activation is involved in antioxidant defenses (Gajewska et al., 2025). This enhanced plant resilience against stress can be ascribable to the rise in these fatty acid compounds. Increased fatty acids promote proper signaling and response by stabilizing cell membranes under stress (Batsale et al., 2021). The decreased production of these metabolites disrupts carbohydrate metabolism and leads to carbohydrate delocalization in response to stress conditions (Molina & Segura, 2021). Based on our findings, abundant production of GDL, L-Cys, GA, and IP in *H. annuus* plants in response to priming is owing to enhanced membrane saturation in priming conditions.

Another issue plants face is metal toxicity. Arsenic toxicity negatively affects plant growth, development, and yield. To reduce its impact on plants, researchers are using several methods. One of them is seed priming by using any ameliorant. In this study, stress decreases plant height and plant dry weight by interfering with the cell’s metabolic machinery. While seed priming with Rib increased plant growth by improving carbohydrate metabolism and energy production. It enhanced the plant development by increasing the cell division and seed germination, ultimately leading to better growth of the plant. Overall, the findings support the hypothesis by presenting better plant growth and development, mitigating the negative impacts of As stress. Also, seed priming with Rib imposed a positive impact on sunflower as compared to hydropriming. These results can be used in future studies for producing metal stress resilient crops of sunflower, and their impact on oil production can be observed.

## Conclusion

Our study concluded that seed priming with 50 ppm riboflavin (Rib) was an efficient method for reducing the effects of arsenic (As) on plant metabolic activity. We found that As hindered plant growth, green pigment synthesis, and gas exchange traits by reducing cell differentiation, increasing ROS generation and electrolyte leakage, ultimately hampering the plant’s growth and defense system. As stress also significantly reduced antioxidants including peroxidase, catalase, and ascorbate peroxidase activity showing a disrupted oxidative balance leading to low scavenging of ROS. However, priming with Rib under no stress conditions increased plant morphological parameters including plant fresh weight, plant height, and plant dry weight. But no significant difference in plant dry weight was observed in plants treated with Rib under As stress. Conversely, in case of plant physiological attributes such as chlorophyll synthesis, photosynthetic rate, gas exchange attributes, and membrane stability index, Rib under As stress played a significant role in improving these traits. The activity of antioxidants was also better in plants treated with Rib under As stress indicating improved stress resilience. This improvement in plant physiology was evident by increased production of certain metabolites such as Choline (Cho) under Rib + As treatment. Choline is the precursor of glycine betaine that helps maintain osmotic balance, membrane stability, ROS homeostasis, and protection of enzymes under abiotic stress. Hence, our findings demonstrate, for the very first time, the use of Rib as a priming agent to enhance the growth of *H. annuus* plants growing in As-contaminated soil. Nevertheless, further studies on vitamin priming on various crops, with different concentrations, biosynthesis pathways, and meta-transcriptomics related to stress tolerance mechanisms are required. This study also suggests that the impact of such priming stimulants and arsenic must be observed through the physicochemical properties of soil as well.

##  Supplemental Information

10.7717/peerj.21596/supp-1 File S1 Supplemental figures and table.

10.7717/peerj.21596/supp-2 Data S1 Raw Data.

## Additional Information and Declarations

### Data Availability

The following information was supplied regarding data availability: The raw data are available in the Supplemental File. The mass spectrometry data is available at Figshare: - Manzoor, Anum; Kamran, Atif; Naveed, Amna; Jahan, Summera; Akram, Waheed; Sardar, Rehana; et al. (2026). GC-MS Data of Sunflower leaf extract studying riboflavin-primed seeds reduce arsenic stress. figshare. Dataset. https://doi.org/10.6084/m9.figshare.32896496
