# Supplementary material for: Riboflavin-primed seeds reduce arsenic stress in Helianthus annuus L. through modulating antioxidant and metabolomics responses
Source: PeerJ. 2026 Oct 6;14:e21596. doi: 10.7717/peerj.21596 (PMC13651778; doi:10.7717/peerj.21596)
Supplement: Supplementary file 1 [file peerj-14-21596-s001.docx]

Figure S1. Pictorial presentation of seedling growth for Riboflavin (Rib) optimization.

| Table S1. Effects of different concentrations of Riboflavin on germination of Riboflavin-primed seeds of *Helianthus annuus* L | | | |
| --- | --- | --- | --- |
| **Treatments** | **Germination rate** | **Germination percentage (%)** | **Germination index** |
| Control (Hydropriming) | 0.78 ^c^ ±0.21 | 25.83 ^d^±1.83 | 2.15 ^c^± 0.31 |
| 25 ppm | 1.16 ^ab^±0.14 | 63.00 ^b^±2.78 | 2.88 ^bc^ ± 0.57 |
| 50 ppm | 1.43 ^a^±0.09 | 96.67 ^a^±3.17 | 3.16 ^a^ ± 0.16 |
| 75 ppm | 1.05 ^abc^±0.17 | 58.00 ^c^±2.07 | 2.33 ^abc^ ± 0.50 |
| Data shows Mean± standard deviation (n=3) | | | |

Graphical abstract

Graphical abstract presenting that seeds are subjected to varying levels of riboflavin (control, 25 ppm, 50 ppm, 75 ppm) and 10 ppm Arsenic (As) to soil. Samples were prepared to observe plant responses to different riboflavin levels under As stress. In the research, several parameters are measured, including physico-biochemical characteristics, photosynthetic content, antioxidant activity, protein content, membrane stability index (MSI), electrolyte leakage (EL), gaseous exchange activity (photosynthetic rate, transpiration rate, stomatal conductance), and phytocompounds using GC-MS.
